# Crystal Structure of Der f 7, a Dust Mite Allergen from *Dermatophagoides farinae*

**DOI:** 10.1371/journal.pone.0044850

**Published:** 2012-09-06

**Authors:** Kang Wei Tan, Chacko Jobichen, Tan Ching Ong, Yun Feng Gao, Yuen Sung Tiong, Kang Ning Wong, Fook Tim Chew, J. Sivaraman, Yu Keung Mok

**Affiliations:** Department of Biological Sciences, National University of Singapore, Singapore, Singapore; National Institute of Environmental Health Sciences, United States of America

## Abstract

**Background:**

Der f 7 is the group 7 allergen from the dust mite *Dermatophagoides farinae*, homologous to the major allergen Der p 7 from *D. pteronyssinus*. Monoclonal antibody that bind to residues Leu48 and Phe50 was found to inhibit IgE binding to residue Asp159, which is important for the cross-reactivity between Der f 7 and Der p 7.

**Methodology/Principal Findings:**

Here, we report the crystal structure of Der f 7 that shows an elongated and curved molecule consisting of two anti-parallel β-sheets – one 4-stranded and the other 5-stranded – that wrap around a long C-terminal helix. The overall fold of Der f 7 is similar to Der p 7 but key difference was found in the β1–β2 loop region. In Der f 7, Leu48 and Phe50 are in close proximity to Asp159, explaining why monoclonal antibody binding to Leu48 and Phe50 can inhibit IgE binding to Asp159. Both Der f 7 and Der p 7 bind weakly to polymyxin B via a similar binding site that is formed by the N-terminal helix, the 4-stranded β-sheet and the C-terminal helix. The thermal stability of Der f 7 is significantly lower than that of Der p 7, and the stabilities of both allergens are highly depend on pH.

**Conclusion/Significance:**

Der f 7 is homologous to Der p 7 in terms of the amino acid sequence and overall 3D structure but with significant differences in the region proximal to the IgE epitope and in thermal stability. The crystal structure of Der f 7 provides a basis for studying the function and allergenicity of this group of allergens.

## Introduction

For decades, the house dust mite has been known as a major causative agent of various allergic diseases including asthma, atopic dermatitis (AD) and allergic rhinitis. The group 7 allergen of dust mite was first isolated from a *D. pteronyssinus* cDNA library and named Der p 7 [1]. Sera from 14/38 (37%) allergic children reacted strongly with Der p 7, and skin prick tests showed reactivity in 16/30 (53%) allergic patients [1]. However, a recent study performed on a total of 253 children in Singapore showed that Der p 7 is a mid-range allergen, with a prevalence of less than 20% as compared with the major allergens, Der p 1 (64%) and Der p 2 (71%) [2]. Recently, the crystal structure of Der p 7 at 2.35 Å was determined as a fusion protein with maltose-binding protein (MBP) at the N-terminus of Der p 7 [3]. Der p 7 has an elongated structure, with two 4-stranded antiparallel β-sheets that wrap around a long C-terminal helix. The fold of Der p 7 is significantly similar to the N-terminal domain of bactericidal/permeability-increasing protein (BPI) [3], which belongs to the PSP/LBP (parotid secretory protein/lipopolysaccharide (LPS) binding protein) superfamily [4]. Der p 7, however, binds to the bacterial lipopeptide polymyxin B (PB) instead of LPS [3].

The homologous allergen, Der f 7, isolated from *D. farinae*, is a 196-residue protein with 86% identity to Der p 7. A Der f 7 fusion protein has been shown to react with IgE antibodies in sera of 19/41 (46%) asthmatic children [5]. Monoclonal antibodies (mAb) produced against Der p 7 and Der f 7 have cross-reacted with the group 7 allergens of both species and blocked IgE binding to these allergens [6], [7], suggesting that Der f 7 and Der p 7 may share similar IgE epitopes. An isoform of Der f 7 has been cloned, expressed and the secondary structure characterized [8], but the detailed three-dimensional structure of Der f 7 is not available. A three-dimensional model of Der f 7 was generated using homology modeling, based on the crystal structure of Der p 7 and used for mAb binding studies [9]. Immunodot blot experiments using overlapping peptides derived from Der f 7 identified Leu48 and Phe50 as the residues that were important for its interaction with HD12, a Der f 7-specific mAb that blocked IgE binding [6], [9]. The corresponding residues on Der p 7, Ile48 and Leu50, do not react with mAb HD12, suggesting that residues Leu48 and Phe50 are unique epitopes in Der f 7 [9]. Subsequently, Chou *et al*. identified residue Asp159, located in the loop region (based on the model of Der f 7), as an important residue that is responsible for the IgE-mediated cross-reactivity between Der f 7 and Der p 7 [10]. This phenomenon, however, was shown in only 2/30 sera tested [10].

As the continuation of our efforts to understand the structure and function of allergens, here we report the crystal structure of Der f 7, its ligand binding and stability when compared with Der p 7. The overall structure of Der f 7 is homologous to Der p 7, except with significant differences in the loop region proximal to the putative IgE epitope residue. Der f 7 binds PB at a similar site as Der p 7 with weak affinity. Der f 7, however, differs significantly in thermal stability as compared to Der p 7 and the stabilities of both proteins are pH dependent. The current study provides the structural basis for studying the allergenicity, function and stability of this group of allergens.

## Results

### Crystal structure of Der f 7

**Figure 1:**
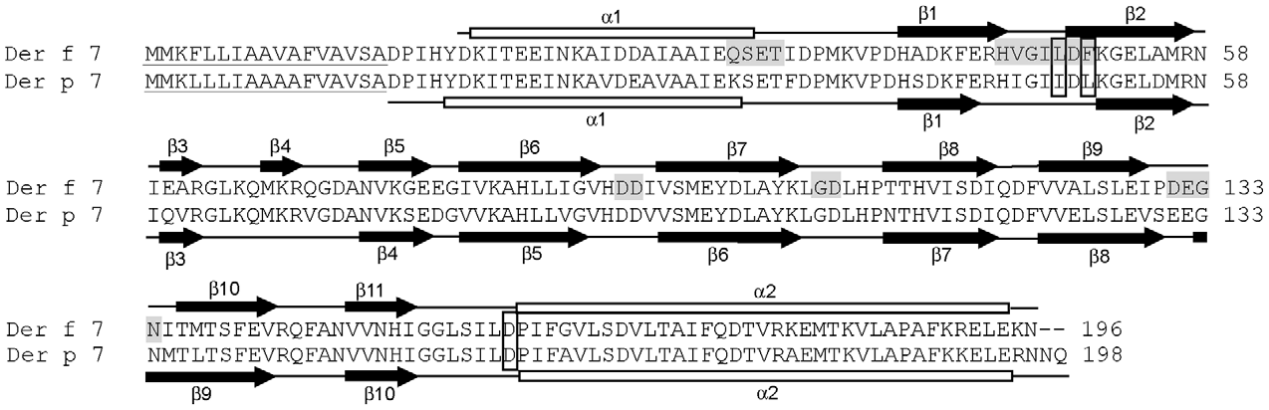
Sequence comparison of Der f 7 with Der p 7. The amino acid sequence of Der f 7 was aligned with Der p 7 using the program ClustalW 2.0.12 [32]. Der f 7 has a sequence identity of 86% as compared with Der p 7. Boundaries of secondary structures as determined from the crystal structures are shown as a cartoon above or below their respective sequences. The IgE binding residues of Der f 7 (Leu48, Phe50 and Asp159) and their corresponding residues in Der p 7 are boxed. Residues that are located at regions with high B-factor are highlighted in grey. Residues that are predicted to be in the signal peptide sequence by the program SIG-Pred are underlined.

We purified a Der f 7 construct (Asp1 to Asn196) and determined its crystal structure by molecular replacement method. In this isoform of Der f 7, residue 130 is a Pro instead of a Ser, as reported in other Der f 7 sequences; the other residues are identical [5], [9] (Fig. 1). The model of Der f 7 was refined up to a resolution of 2.0Å with R-factor of 0.224 (R_free_  = 0.28) (Table 1). The electron density for the N-terminal residues from Asp1 to Tyr5 and the C-terminus residue Asn196 were disordered and these residues were not included in the model. Furthermore, the electron density for the side chain of residues Lys7 (α1), Lys51 (β2), Glu79 (β5–β6 loop), His91 to Asp93 (β6–β7 loop), Asp131 to Asn134 (β9–β10 loop) and Arg191 were not well defined and were therefore only modeled with backbone and sometimes Cβ atoms. Glu27 and Ile47 are in the disallowed region of the Ramachandran plot, and both residues are located in the loop regions and were well defined in the electron density map. In the crystal structure of Der p 7, it was reported that the regions Gly46 to Leu50 and Glu42 to Leu50 (β1–β2 loop) were missing in chain B and C, respectively. The observed flexible regions are different in the structures of Der f 7 and Der p 7 [3], suggesting that these structures are complementary to provide structural information on this group of allergens.

**Table 1:** Crystallographic data statistics of the structure of Der f 7.

Wavelength (Å)	1.5418
Oscillation angle (°)	0.5
Space group	P2_1_2_1_2_1_
Unit-cell parameters	
* a* (Å)	51.09
* b* (Å)	58.23
* c* (Å)	128.98
Resolution limits (Å)	50.0–1.90
Observed *hkl*	326532
Unique *hkl*	30707
Redundancy	10.6
Completeness (%)	98.4
Overall *l*/σ (*l*)	6.7
Rsym	0.11
Refinement statistics	
Rcryst	0.224
Rfree	0.280
Overall mean B value (Å^2^)	33.85
Number of water	281
Root mean square deviation from ideal values	
Bond length (Å)	0.009
Bond angle (°)	1.188
Ramachandran plot statistics	
Residues in most favored regions (%)	90.4
Residues in additionally allowed regions (%)	8.4
Residues in generously allowed regions (%)	0.3
Residues in disallowed regions (%)	0.9

Rsym  =  Σ |*Ii – <I>*|/Σ |*Ii*|, where *Ii* is the intensity of the *i*th measurement and *<I>* is the mean intensity for that reflection.

Rcryst  =  Σ ||Fo| – |Fc||/Σ |Fo| calculated from the working data set.

Rfree was calculated from 5% of data randomly chosen not to be included in refinement.

Ramachandran results were determined by using PROCHECK.

**Figure 2:**
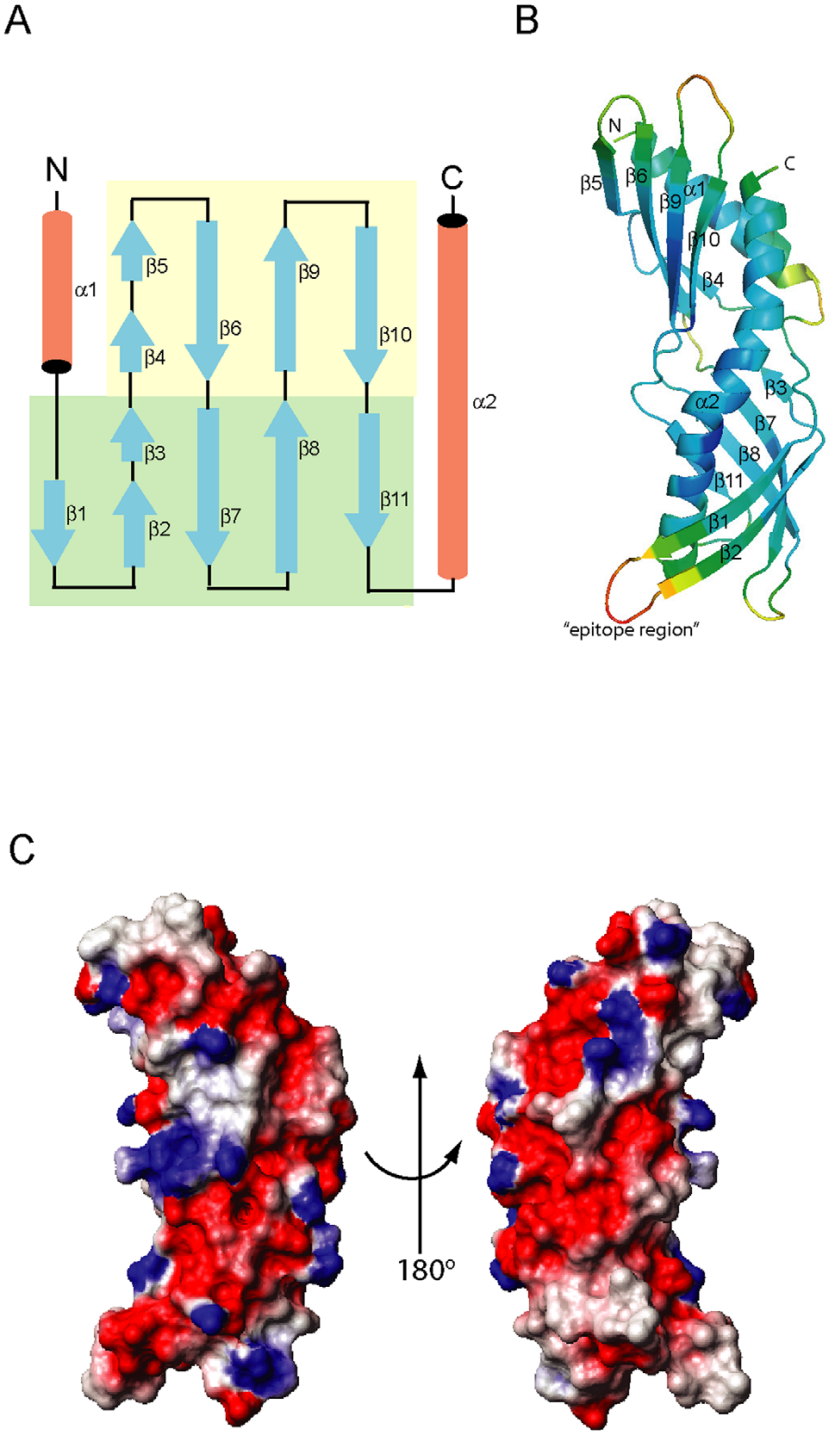
Crystal structure of Der f 7. (A) Topology diagram of Der f 7. The 4-stranded β-sheet is colored yellow and the 5-stranded β-sheet is colored green. (B) Ribbon diagram of Der f 7 is colored based on the B-factor spectrum, with a range from 13.59 (blue) to 70.52 (red). The loop region between β1 and β2 is labeled as the “epitope region”. The figure was generated using the program PyMol [33]. (C) Two different views of the surface charge diagram of Der f 7. The positively and negatively charged residues are colored blue and red, respectively. The diagram was generated using the program Molmol [34].

In the crystal structure of Der f 7, the 11 β-strands are arranged to form an elongated, curved anti-parallel β-sheet that wraps around the long C-terminal helix, α2; this is compared with the 10 β-strands in Der p 7. The single β-sheet has loops at the middle of each β-strand, making it appear as if the two β-sheets are joined together. The 4-stranded β-sheet comprised β-strands β4/β5, β6, β9 and β10, whereas the 5-stranded β-sheet comprised β-strands β1, β2/β3, β7, β8 and β11 (Fig. 2A). The N-terminal helix, α1, interacts closely with the 4-stranded β-sheet and the C-terminal end of the helix α2 to form a hydrophobic channel, likely for ligand binding (Fig. 2B). A search for structurally similar proteins using DALI revealed that the Der f 7 structure is homologous to the following structures in descending order, based on the Z-score: MBP-Der p 7 fusion protein (3H4Z, Z-score 26.9) [3]; juvenile hormone binding protein (JHBP) from *Galleria mellonella* hemolymph (2RCK, Z-score 13.3) [11]; human bactericidal permeability-increasing protein (BPI) (1BP1, Z-score 12.7) [12]; JHBP from silk worm in complex with JH3 (2RQF, Z-score 11.9); JHBP from silk worm (3A1Z, Z-score 11.8); BPI (1EWF, Z-score 11.3) [13]; *Epiphyas postvittana* Takeout 1 (3E8W, Z-score 11.2) [14]; and cholesteryl ester transfer protein (2OBD, Z-score 10.6) [15]. All these proteins engage ligand of hydrophobic nature, suggesting that Der f 7 may also be involved in binding of hydrophobic ligand.

Based on B-factor distribution of Der f 7 (Fig. 2B), the loop region between β1 and β2 showed the highest B-factor. This region (HVGILDF, from residues His44 to Phe50) harbors the proposed IgE epitope of residues Leu48 and Phe50 [9]. The residue Asp159, a proposed IgE epitope important for the cross-reactivity between Der f 7 and Der p 7 [10] is located at a region with low B-factor value. The other loop region with a relatively high B-factor is that between β9 and β10 (DEGN, residues Asp131 to Asn134); this is also disordered in the electron density map and indicates its flexibility. This loop between β9 and β10 is the only region that is flexible in Der f 7, yet it appears rigid in Der p 7 based on B-factors of Der p 7. Another region of Der f 7 with a relatively high B-factor comprises residues Gly106 and Asp107 between β7 and β8; this loop is spatially right next to the loop between β1 and β2 (Fig. 2B). The QSET sequence (residues Gln25 to Thr28) between α1 and β1, and residues Asp92 and Asp93 between β6 and β7 are also considered relatively flexible, with high B-factor values.

**Figure 3:**
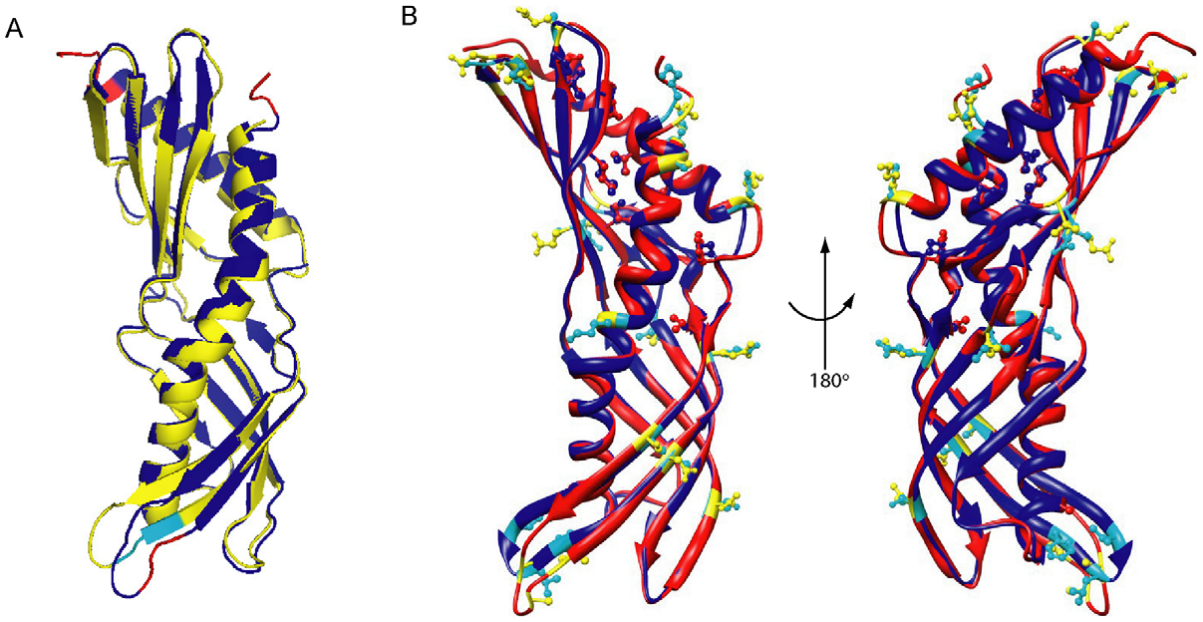
Comparison of the structures of Der f 7 and Der p 7. (A) Superposition of the crystal structures of Der f 7 and Der p 7 prepared using the TopMatch web service [35], [36]. The root-mean-square-error of superposition of Der f 7 and Der p 7 is 0.97 Å for 186 structurally equivalent Cα atoms. Der f 7 is colored cyan and Der p 7 is colored red. Pairs of structurally equivalent residues are colored yellow (Der f 7) and blue (Der p 7). The figure was generated using PyMol [33]. (B) Non-conserved residues between Der f 7 (blue) and Der p 7 (red) are shown as ball-and-stick models on the superimposed structures of Der f 7 and Der p 7. Non-conserved residues that are also surface exposed are colored cyan (Der f 7) and yellow (Der p 7), including Asp18/Glu18, Gln25/Lys25, Ala38/Ser38, Val45/Ile45, Leu48/Ile48, Phe50/Leu50, Ala55/Asp55, Glu60/Gln60, Gln70/Val70, Gly77/Ser77, Glu79/Asp79, Ile81/Val81, Ile94/Val94, Thr111/Asn111, Ala124/Glu124, Asp131/Glu131, Lys179/Ala179, Arg191/Lys191 and Lys195/Arg195, respectively. The figure was generated using Chimera [37].

**Figure 4:**
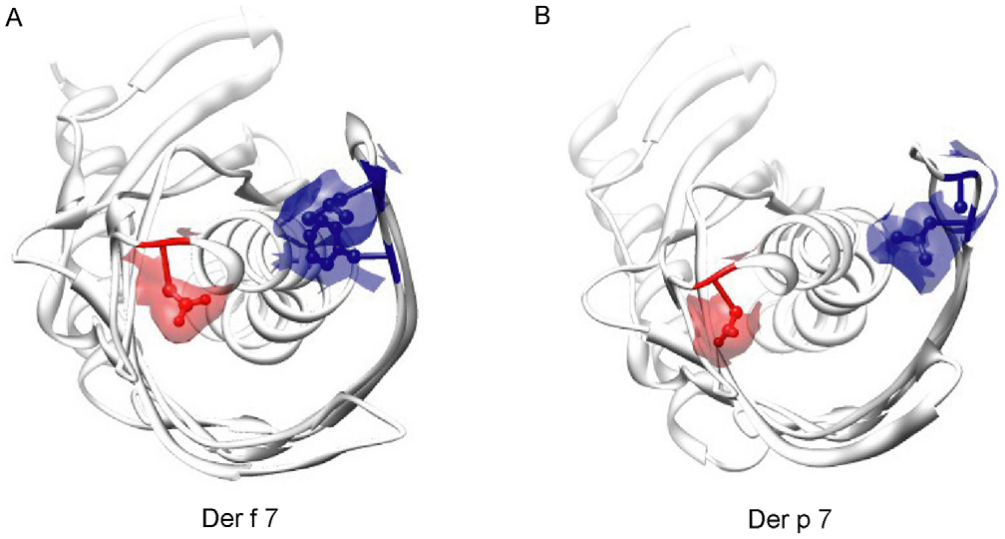
IgE epitopes of Der f 7. Ribbon diagrams of (A) Der f 7 and (B) Der p 7 showing locations of the putative IgE binding epitope residues of Der f 7, Leu48, Phe50 and Asp159, and their corresponding locations on Der p 7. These residues were plotted as both ball-and-stick and surface models with 50% transparency. Residues 48 and 50 are colored blue, while residue 159 is colored red. The diagrams were generated using the program Chimera [37].

The surface of Der f 7 consists of many charged amino acids, typical of an inhalant allergen (Fig. 2C). The ExPASy predicted isoelectric points of Der f 7 and Der p 7 as 4.90 and 4.85, respectively. Superposition of Der f 7 and Der p 7 showed that, with the exception of the N- and C-termini, the only region that is not structurally equivalent lies at the loop between β1 and β2, which harbors the largest conformational difference between these two proteins (Fig. 3A). Notably, this loop contains the linear epitope residues, Leu48 and Phe50, for the mAb HD12 that was found to inhibit IgE binding to Der f 7 [9]; these mAb epitope residues are unique to Der f 7 and found to be replaced with Ile48 and Leu50 in Der p 7. The other non-conserved residues between Der f 7 and Der p 7 are mainly exposed residues and located at the other end of the elongated molecule, opposite to this loop region (Fig. 3B). The putative IgE epitope residue of Der f 7 and Der p 7, Asp159, is located at the start of helix α2 and in proximity of the β1–β2 loop. This distance from Asp159 to Leu48 and Phe50, however, is highly different in Der f 7 and Der p 7. In Der f 7, Asp159 is located closely to the β1–β2 loop with a distance of only 7.7Å between Asp159 OD1 and Phe50 CZ (Fig. 4A). In contrast, the distance between Asp159 OD2 and Leu50 CD2 is 14.3Å in Der p 7 (Fig. 4B). This structural difference may explain the difference in behaviors of mAb and IgE binding to this region of the two proteins.

### Ligand binding by Der f 7

**Figure 5:**
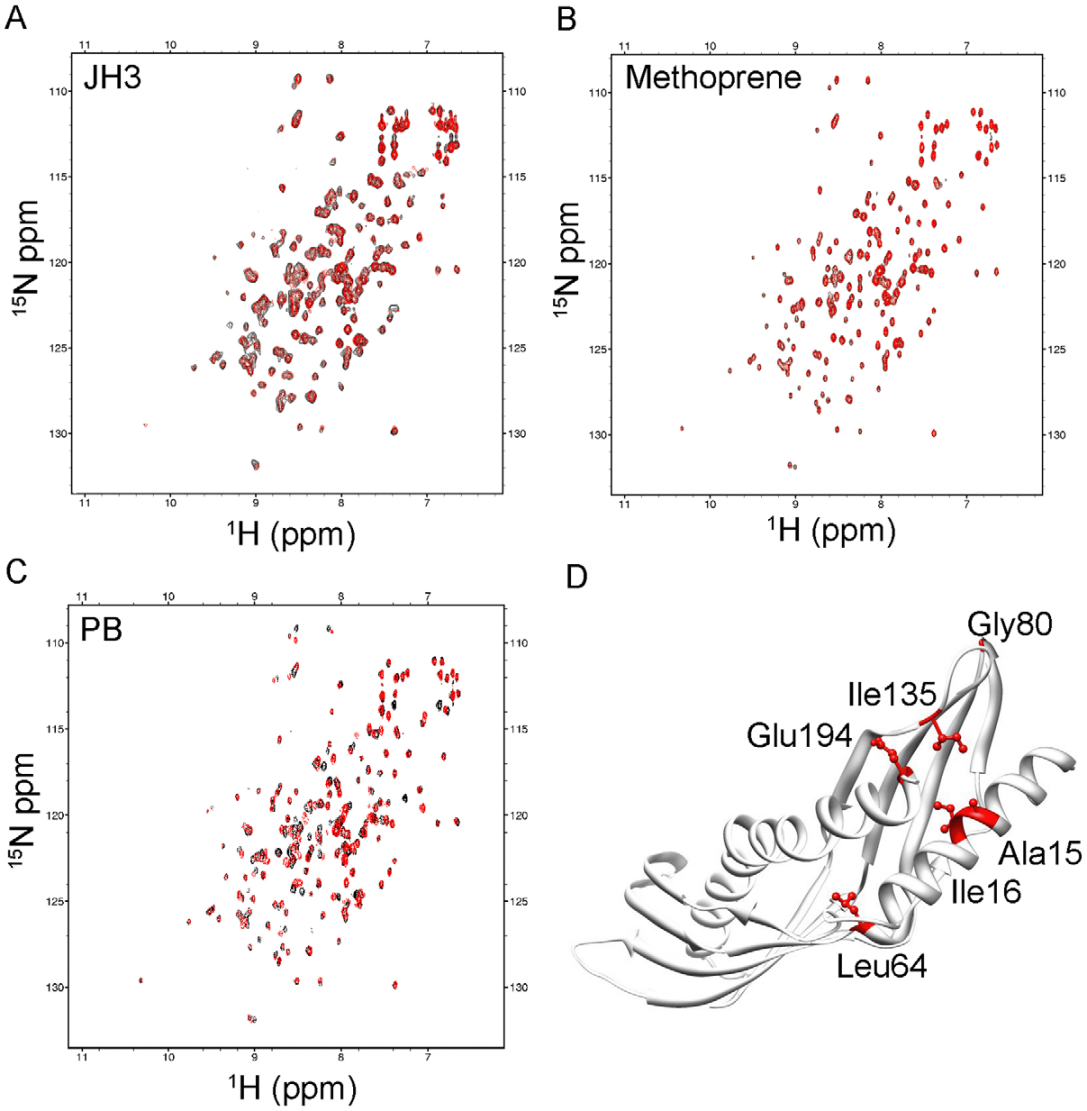
Ligand binding of Der f 7. ^1^H-^15^N HSQC spectra of 0.5 mM Der f 7 in the absence (black) or presence (red) of (A) juvenile hormone III (JH3); (B) methoprene; and (C) polymyxin B (PB) at a molar ratio of 1∶1 (protein:ligand). (D) Ribbon diagram of Der f 7 showing locations of residues that had their combined NH and ^15^N chemical shift values perturbed by more than 0.3 ppm in the presence of PB. These residues are presented in ball-and-stick models and colored red. The figure was generated using the program Chimera [37].

Previously, it was reported that Der p 7 does not bind lipopolysaccharide (LPS) but binds bacterial lipopeptide polymyxin B (PB) with weak affinity in the predicted binding site of Der p 7 [3]. Here, we show the binding of Der f 7 to three different types of putative ligands: PB, JH3 and methoprene. Methoprene is a juvenile hormone (JH) analog that regulates insect growth and effectively suppresses population growth of the dust mite *D. farinae* [16]. Our results showed that Der f 7 does not bind to JH3 or methoprene, but binds weakly to PB (similar to Der p 7) based on a chemical shift perturbation in ^1^H-^15^N HSQC NMR spectrum of Der f 7 following titration of ligands (Fig. 5A –5C). The residues perturbed by more than 0.3 ppm (combined ^15^N and NH chemical shift) were Ala15, Ile16, Leu64, Gly80, Ile135 and Glu194 (Fig. 5D). These residues are located around a hydrophobic cleft formed between the N-terminal helix α1 (Ala15 and Ile16), the 4-stranded β-sheet (Leu64, Gly80 and Ile135), and the C-terminal helix α2 (Glu194). Most of these residues are hydrophobic in nature, suggesting that hydrophobic interactions play an essential role in ligand binding. These residues perturbed by PB binding on Der f 7 are located at a similar binding site as that which was proposed for Der p 7 [3].

### Stabilities of Der f 7 and Der p 7 at different pH values

**Figure 6:**
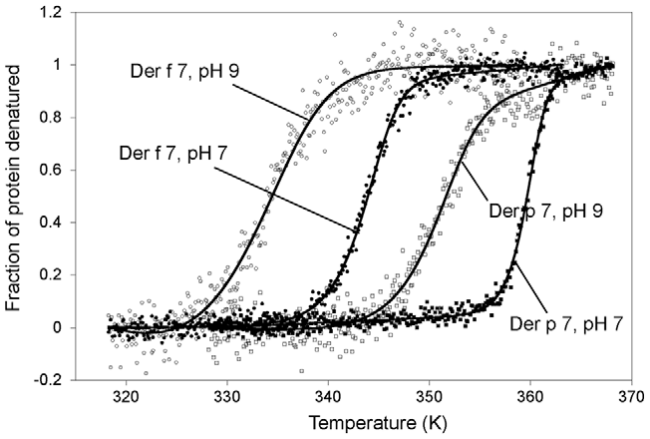
Thermal stabilities of Der f 7 and Der p 7 at different pH values. Thermal denaturation of Der f 7 at pH 7.0 (closed circles) or pH 9.0 (open circles) and Der p 7 at pH 7.0 (closed squares) or pH 9.0 (open squares) were monitored by Far-UV CD signals at 220 nm. The fitted curves are shown as solid lines through the corresponding data points.

In addition to the lack of cross-reactivity between Der f 7 and Der p 7, we also found significant differences in their stabilities, both of which are highly depend on the pH of the buffer, as determined based on thermal denaturation and Far-UV CD monitoring. The T_m_ for thermal denaturation values of Der f 7 at pH 7.0 and pH 9.0 were 73.8°C and 65.7°C, respectively; whereas the T_m_ for thermal denaturation values of Der p 7 at pH 7.0 and pH 9.0 were 88.4°C and 81.6°C, respectively (Fig. 6). In general, Der p 7 shows more thermal stability than Der f 7, by a difference in T_m_ of around 15°C and both proteins are more stable at pH 7.0 than 9.0, by a difference in T_m_ of around 7°C. This significant drop in thermal stability of Der f 7 was not because of the Pro130 residue found in this particular construct, as a similar thermal stability was found when we mutated residue Pro130 to Ser (Fig. S1).

## Discussion

The crystal structure of Der f 7 is highly homologous to proteins of the PLUNC family [17], which have hydrophobic ligand binding properties. A DALI search for homologous structures of Der f 7 showed that, other than Der p 7, JHBP is the most structurally related to Der f 7. Insect JHBP is essential for transporting the juvenile hormone (JH) in the hemolymph of butterflies and moths [11]. JHBP possesses two hydrophobic cavities at opposite ends of the molecule and binds JH in one of the cavities closest to the N- and C-termini [11]. Der f 7 also showed significant homology to the N-terminal domain of BPI, a protein with potent bactericidal activity against gram-negative bacteria via its interaction with the lipid A portion of LPS [18]. BPI has two apolar pockets on the concave surface of the boomerang-shaped molecule, each of which binds a molecule of phosphatidylcholine (POPC). For the N-terminal barrel, POPC is bound in a pocket, with the entrance formed by helix A and helix B [12]; this is similar to the pocket formed by the N-terminal helix α1 and the C-terminal helix α2 in Der f 7. The other insect-related protein showing high homology with Der f 7 is the *Epiphyas postvittana* takeout protein [14]. This protein has a 45 Å-long, purely hydrophobic internal tunnel that extends for the full length of the protein and accommodates a hydrophobic bound ligand [14]. The high structural homology of Der f 7 with these proteins suggests that Der f 7 may also be involved in the binding and transportation of similar hydrophobic ligands essential for development or innate immune defense in dust mites.

Significant structural difference was observed between Der f 7 and Der p 7 in the β1–β2 loop region, which contains the linear epitope residues, Leu48 and Phe50, of the Der f 7 specific mAb HD12 [9]. The mAb HD12 was found to block IgE binding to Der f 7 [6]. In the studies by Shen H–D. *et al.* and Chou H. *et al.*, peptide (Df7–5) derived from the β1–β2 loop region of Der f 7 can inhibit mAb binding to Der f 7, but this peptide cannot inhibit IgE binding to Der f 7 [9], [10]. In addition, peptide (Df7–16) derived from the region of the putative IgE epitope residue, Asp159, can inhibit IgE binding to Der f 7 but cannot inhibit the binding of mAb HD12 to Der f 7 [9], [10]. This suggests that mAb HD12 and IgE occupy two separate linear epitopes on Der f 7. Based on the crystal structure of Der f 7, the distance between these two linear epitopes is very close (Fig. 4A). This explains why the binding of mAb HD12 can disrupt IgE binding to Der f 7, likely due to steric hindrance between the mAb and IgE. The mAb HD12 is specific to Der f 7 and should not bind Der p 7 in which residues Leu48 and Phe50 are being replaced with Ile48 and Leu50 [6], [9]. The above results showed that the indirect method of using mAb, which can disrupt IgE binding to an allergen, for IgE epitope mapping may not be able to pinpoint the exact location. Direct method based on inhibition of IgE binding using site-directed mutants of the allergen should be used to verify the mapped IgE epitopes.

To locate the exact position of the IgE epitope in Der f 7, Clhou *et al.* used direct IgE binding method instead of the indirect mAb method. D159A mutant of Der f 7 has a lower IgE reactivity and cannot inhibit IgE binding to Der f 7 to the same degree as wild type Der f 7 [10]. Hence, Asp159 was proposed as a linear IgE epitope of Der f 7. In the same study, Der p 7 was found to cross-react with Der f 7 based on the observation that a peptide derived from the region of Asp159 in Der p 7 can inhibit the binding of IgE to Der f 7 [10]. In addition, the D159A mutant of Der f 7 has significantly reduced inhibition of IgE binding to Der p 7, but the wild type Der f 7 can inhibit IgE binding to Der p 7 at a similar degree as Der p 7 itself [10]. These results suggest that Der p 7 may also contain a linear epitope at the region of Asp159, which is highly conserved in terms of sequence and structure between Der p 7 and Der f 7. However, the degree of IgE binding of Der f 7, in the study region was only around 30% of that of Der p 7, corresponding to the strong bias of *D. pteronyssinus* infestations over infestation with *D. farinae*. Further study of the Der f 7 epitopes and cross-reactivity needs to include the examination of sera from subjects predominantly exposed to *D. farinae*.

It has been shown that Der p 7 binds weakly to PB, but not to LPS [3]. As Der f 7 is homologous to Der p 7 and JHBP, we assigned the ^1^H-^15^N-HSQC NMR spectrum of Der f 7 and determined the binding of PB, JH3 and methoprene to Der f 7, based on chemical shift perturbation. PB is a natural peptide and a potent antibiotic that binds to and neutralizes LPS [19]. JH is responsible for initiation of vitellogenesis and female reproduction in most insects. However, in the Acari, including both ticks and mites, it seems that ecdysteroids, not JH, regulates vitellogenesis [20]. Methoprene is a non-terpenoidal JH analog that resembled the basic terpenoid structure of JH and has been shown to suppress population growth of dust mites [16], [21]. Der f 7 and Der p 7 are structurally homologous to JHBP, BPI and LPS binding protein. However, instead of binding to JH3 or LPS, Der f 7 and Der p 7 bind to PB, which is the molecule shown to interact with LPS. Whether PB is a natural ligand of Der f 7 and the physiological relevance of PB in dust mite remain to be determined. The IgE epitope is located on the opposite end to the ligand binding site of the elongated molecule; as such, there is a very low chance of ligand binding to affect the allergenicity of Der f 7.

Although Der f 7 and Der p 7 both interact with the same ligand, their stabilities are found to be significantly different and highly depend on the pH of the buffer. The correlation between the allergenicity, function and stability of Der f 7 and Der p 7 is not clear. The recombinant Der f 7 is much less stable than Der p 7 at both pH 7.0 and pH 9.0 showing that it is important to compare the results of the IgE binding activity to Der p 7 by subjects sensitized by *D. pteronyssinus* to the IgE binding of Der f 7 by sera from subjects exposed to *D. farinae*. Further studies examining the stability and allergenicity of allergens are required to establish a correlation, if any exists.

### Conclusions

In summary, Der f 7 is homologous to Der p 7 in terms of the amino acid sequence and 3D structure. However, there are significant differences in the β1–β2 loop region proximal to the putative IgE epitope residue. Both Der f 7 and Der p 7 weakly bind to PB in a similar binding site, but their thermal stabilities are different and highly depend on pH. The crystal structure of Der f 7 provides a basis for studying the allergenicity, function and stability of proteins in this group of allergens.

## Materials and Methods

### Expression and purification of Der f 7

Der f 7 was sub-cloned, expressed and purified as a His-tag labeled protein using Ni-NTA affinity column (Qiagen) followed by a Superdex-200 gel filtration column (GE Healthcare). The details of expression and purification of Der f 7 are given in Tan *et al.*, 2011 [22].

### Crystallization and structure determination

Prior to crystallization, purified Se-Met labeled Der f 7 protein was dialyzed into 10 mM HEPES buffer at pH 7.0 and concentrated to 8 mg/ml with an Amicon filter (Millipore, 10 kDa molecular weight cut-off). The diffraction quality crystal was obtained after 1 week from the reservoir solution containing 0.1 M Bis-Tris pH 7.4 and 28% polyethylene glycol monomethyl ether (PEG MME) 2000. The complete diffraction data was collected using the in-house Bruker MICROSTAR X-ray generator equipped with a PLATINUM 135 CCD detector. Subsequent data set processing was done using the *HKL-*2000 program suite [23]. The structure was solved by molecular replacement method using PHASER program from the CCP4 suite [24]. Der p 7 (PDB code 3H4Z, sequence identity  = 86%) was used as the search model. Model building was performed using the COOT program [25] and refinement was carried out using PHENIX [26].

### Far-UV circular dichroism (CD) and thermal denaturation

Far-UV CD experiments were carried out with 15 μM protein (Der f 7 and Der p 7) in 50 mM Tris-HCl at different pH levels (pH 7 and pH 9). The spectra were acquired with a J–810 Spectropolarimeter (Jasco, Japan) using a quartz cuvette with 1 mm path length (Hellma, Germany). Thermal denaturation experiments were carried out for Der f 7 and Der p 7 by recording the molecular ellipticity at 220 nm for which the change was most drastic with temperature increments. Spectra were recorded at the temperature slope of 1°C/min with the resolution of 0.1°C across a range of 55–95°C. T_m_ was determined by curve fitting according to the equation by Ruiz-Sanz *et al.* [27] using the program KaleidaGraph.

### Assignment of HSQC spectrum for ligand binding site determination

3D heteronuclear NMR experiments were carried out to assign the backbone chemical shifts using a 2D ^1^H-^15^N HSQC as a reference spectrum. All NMR experiments were carried out on a Bruker AVANCE 800 MHz spectrometer equipped with a cryoprobe. Samples were loaded into a 5 mm NMR tube and all experiments were carried out at 40°C. All data acquired were processed using NMRPipe [28] and viewed and analyzed using NMRDraw [28] and SPARKY [29]. Computational processing was carried out on Dell Precision 360 workstation and SGI Origin 300 server. The 2D ^1^H-^15^N HSQC spectrum was acquired with 1280 complex points over a spectral width of 11160.7 Hz on the amide proton dimension. In the ^15^N dimension, 128 points were collected over 1865 Hz. Carrier frequencies for ^1^H and ^15^N were at 4.7 ppm and 117.5 ppm, respectively. Backbone assignments were carried out using HNCACB [30] and CBCA(CO)NH [31] experiments. In the acquired F1 dimension (^1^H) of the experiment, 1280 points were collected over a spectral width of 11160.7 Hz. In the F2 dimension (^15^N), 92 points were collected over 1865 Hz of spectral width, while the F3 dimension (^13^C) contained 116 points covering 12072.4 Hz spectral width. The carrier frequencies for F1, F2 and F3 dimensions were at 4.7 ppm, 118.5 ppm and 41.7 ppm, respectively. The 3D CBCA(CO)NH experiment was carried out under similar conditions, using the same data points and spectral widths as above, except that the F2 dimension (^15^N) was recorded with 40 points. For ligand binding studies, the highly purified Der f 7 was dialyzed into 50 mM Tris buffer at pH 7.0 and concentrated to 0.5 mM. 2D ^1^H-^15^N HSQC spectra were acquired with four scans using similar parameters as described earlier, with and without the addition of PB, insect juvenile hormone III (JH3) or methoprene at 1∶1 molar ratios. Perturbation of peaks by ligand titration was estimated using the formula: √(ΔH)^2^+(ΔN*0.17)^2^ and plotted against residue numbers.

### Data deposition

The crystal structure of Der f 7 has been deposited at the Protein Data Bank with accession ID of 3UV1.

## Supporting Information

Figure S1 **Thermal stabilities of wild type and P130S mutant of Der f 7 at different pH values.** Thermal denaturation of Der f 7 at pH 7.0 (closed circles) or pH 9.0 (closed squares) and P130S mutant of Der f 7 at pH 7.0 (open squares) or pH 9.0 (open circles) were monitored by Far-UV CD signals at 220 nm.
(TIF)
